# Task shifting levonorgestrel implant insertion to community midwife assistants in Malawi: results from a non-inferiority evaluation

**DOI:** 10.1186/s40834-018-0077-6

**Published:** 2018-11-01

**Authors:** Dylane N. Davis, Clara Lemani, Nenani Kamtuwanje, Billy Phiri, Prisca Masepuka, Sally Kuchawo, Nivedita L. Bhushan, Jennifer H. Tang

**Affiliations:** 1 UNC Project-Malawi, c/o Kamuzu Central Hospital, 100 Mzimba Road, Private Bag, A-104 Lilongwe, Malawi; 2 Department of Obstetrics & Gynecology, University of North Carolina at Chapel Hill, 100 Manning Drive, Chapel Hill, North Carolina, 27599-7570 CB-7570 USA; 3 Department of Health Behavior, University of North Carolina at Chapel Hill, Rosenau Hall, CB #7440, Chapel Hill, North Carolina, 27599-7570 27599 USA

**Keywords:** Contraception, Nurses, Assessment, Developing countries, Family planning, Health workers

## Abstract

**Background:**

In 2013, Malawi began task shifting long acting reversible contraception (LARC) insertion from Nurse Midwife Technicians (NMTs), who undergo 3 years of training, to Community Midwife Assistants (CMAs), who undergo 18 months of training. However, there is no evidence on whether CMAs have the same competency as NMTs for LARC insertion. Therefore, we describe a non-inferiority evaluation to determine whether CMAs are non-inferior to NMTs for the insertion of levonorgestrel (LNG) contraceptive implants in Malawi.

**Methods:**

One CMA and one matched NMT from 31 health centers across Malawi were selected for training in Malawi’s 1-week LARC insertion course in October 2016, and 31 CMAs and 30 NMTs completed the training. After the course, two Family Planning Master Trainers visited the nurses’ health centers over a 5-month period and used the Malawi LNG implant insertion checklist to evaluate the first five LNG implant insertions that each nurse performed during the monitoring visit. A non- inferiority margin of 10% was used to compare mean implant scores between CMAs and NMTs.

**Results:**

We were able to fully evaluate 29 CMAs and 29 NMTs with the LNG implant insertion checklist. The CMAs and NMTs had mean scores of 90.2% and 89.7%, respectively, which were non-inferior (mean difference − 0.5%; 95% CI -3.4%, 2.4%), even when adjusted for the number of years post-graduation and the number of LNG implants inserted pre-training, during training, and since training (mean difference 1.3%; 95% CI -2.2%, 4.8%).

**Conclusions:**

CMAs were non-inferior to NMTs with LNG implant insertion, and both cadres were generally observed to be competent with their insertions after completing their follow-up evaluations. During the evaluations, we generally saw an increase in scores with each insertion. Therefore, for both cadres, it is important to establish continued mentorship and evaluation for LARC insertion after the initial training.

**Electronic supplementary material:**

The online version of this article (10.1186/s40834-018-0077-6) contains supplementary material, which is available to authorized users.

## Background

Widespread shortages in healthcare providers have led to uneven distribution of healthcare access, particularly in rural settings and in countries with limited resources [1]. To circumvent this challenge, many countries have created new health cadres with fewer years of training so that more providers can be quickly trained and improve access to health services [2]. Task shifting various responsibilities and procedures to these new cadres has been proposed as a way of offloading tasks and roles from more highly trained providers so that they can focus more on the difficult and specialized cases and procedures [3]. A particular emphasis has been made on task shifting maternal and newborn health care, such as family planning (FP), to improve maternal and neonatal health access and outcomes [4].

According to the WHO, there is a current estimation of 3420 bedside midwives in Malawi, a population to midwife ratio of 5058:1, which is far higher than the recommended population to midwife ratio of 175:1 [5]. An additional 20,217 bedside midwives are needed to sufficiently address the country’s health needs [5]. Malawi has thus created various auxiliary health cadres, such as Nurse Midwife Technicians (NMTs) and Health Surveillance Assistants (community health workers known as HSAs), to supplement the traditional cadres of Registered Nurses and Registered Nurse Midwives.

The newest auxiliary health cadre created in Malawi is the Community Midwife Assistant (CMA). CMAs receive 18 months of training post-secondary school and receive a certificate after completion of their studies [6]. In comparison, the NMTs receive 3 years of training and receive a diploma after completion. In Malawi, NMTs deliver the majority of FP services. They are trained to provide both short-acting and long-acting reversible contraception (LARC). However, with the growing need of personnel to deliver FP services to patients, task shifting such services from NMTs to CMAs has been considered as a potential strategy to fulfill the growing population’s FP needs, and CMAs were approved to insert and provide LARC in 2013.

To help countries develop and implement task shifting programs, the WHO developed a guidance document in 2012 with “evidence-based recommendations to facilitate universal access to key, effective maternal and newborn interventions,” including recommendations for FP [4]. While the WHO recommends that Registered Nurses and Diploma Nurses (such as NMTs) should be allowed to insert and remove contraceptive implants, they recommend that Auxiliary Nurse Midwives (such as CMAs) should only insert and remove implants “with targeted monitoring and evaluation” [4]. This recommendation was made because there is limited evidence about the effectiveness of allowing Auxiliary Nurse Midwives to insert and remove implants. There is currently no published evidence as to whether CMAs are competent at inserting and removing implants.

### Intervention description

In light of the limited evidence for Auxiliary Nurse Midwives such as CMAs to insert contraceptive implants, we conducted a non-inferiority study to assess whether CMAs were competent at inserting the 5-year levonorgestrel (LNG) implants and whether they had similar competency as NMTs with inserting LNG implants in Malawi.

## Methods

### Study design

There were 200 CMAs selected by their communities, the Malawi Safe Motherhood Initiative Program, and UNC and financially sponsored for training in Malawi between 2014 and 2016 in part from the authors’ institute and a foundation. Of these 200 CMAs, 31 were selected by the Malawi Ministry of Health (MoH) for deployment in October 2016 to a rural health center in the district from which they originated. For our study, we planned to train these 31 CMAs in the 1-week MoH-approved LARC insertion course in October 2016, which was provided in addition to their prior CMA training. During these trainings, we also planned to train 31 matched NMTs, one each from the same rural health center that each CMA was to be deployed.

The LARC training involved two days of didactics and then three days of practical sessions in a government health center. Each training had approximately 15 trainees and 3 trainers as per MoH guidelines. After the training, the CMAs and NMTs returned to their assigned health center. Two UNC Project-Malawi NMT Master FP Trainers then traveled to each of the health centers to evaluate the trained CMAs and NMTs in LNG implant insertion using the MoH-approved LNG Implant Insertion Checklist (Additional file 1) from November 2016 to March 2017. We were unable to evaluate nurse competency for LNG implant removal, etonorgestrel (ENG) implant insertion and removal, and intrauterine device insertion and removals due to the minimal request in the clinics for these contraceptives. The LNG Implant Insertion Checklist consists of a total of 63 assessments for a total of 63 points and includes assessment of pre-insertion counseling, screening, and preparation; insertion technique; and post-insertion tasks, including post-insertion counseling. For each CMA and NMT that could be reached during our evaluation time period, our two Master FP Trainers evaluated the first five LNG implant insertions that occurred during their monitoring visit.

### Sample size calculation

The Malawi MoH deems competency in LNG implant insertion if the trainee has scored at least 85% on the LNG Implant Insertion Checklist. Therefore, for our sample size calculation, we assumed that the NMTs would have a mean LNG insertion score of 95%. We then set the non-inferiority margin at 10% so that the CMAs would still be above the 85% minimum score to meet MoH standards if they were non-inferior. To detect non-inferiority with 90% power and a one-sided alpha of 0.025, we estimated that a sample size of 200 (100 per group) would be needed. We then adjusted for clustering to account for multiple observations for the same individual. Specifically, each trained NMT and CMA would serve as their own cluster for their five scores. Using an expected intra-class correlation of 0.05, we found that we needed to have at least 115 LNG implant insertions in each group, or at least four LNG insertions per nurse if all 62 pre-selected nurses were evaluated. To account for potential loss-to-follow-up among our 62 nurses, we opted to evaluate 5 insertions per nurse to ensure that we had at least 115 insertions in each group.

### Data analysis

Fisher’s exact test was utilized to compare baseline characteristics between the CMAs and NMTs. We then averaged the first five evaluated scores of each CMA and each NMT to determine if each cadre was competent (mean score > 85%) for LNG implant insertion. Generalized linear models were used to evaluate if CMAs had a mean LNG implant insertion score that was within the 10% non-inferiority margin of the NMT LNG implant insertion score. Non-inferiority (NI) would be demonstrated if the lower confidence limit for the difference in mean percent of average implant insertion scores between the two cadres of nurses lay above -Δ NI = − 10. We ran both an unadjusted and adjusted models to account for the number of years since graduation and number of LNG implant insertions performed pre-training, during training, and since training by the end of their monitoring visit. The sample size and data analysis calculations were all performed using STATA, Version 11 (College Station, TX, USA).

## Results

### Nurse characteristics

During our LARC training in October 2016, we trained all 31 of our UNC-sponsored CMAs and 31 other health providers (29 NMTs, 1 Senior Nursing Officer, and 1 Medical Assistant) from each of their health centers. Two health centers did not have an NMT who had been previously trained in LARC insertion before, so they sent other qualified providers (a Senior Nursing Officer and a Medical Assistant, both of whom are considered qualified to place LNG implants in Malawi) instead for the training. In addition, one of the 31 pre-selected NMTs did not show up for the training.

During the 5-month evaluation period (November 2016–March 2017), we were able to evaluate at least 5 LNG implant insertions on 93.5% (29 of 31) of the CMAs and 96.7% (29 of 30) of the other trained health providers. We were unable to assess 1 CMA and 1 NMT due to travel conditions, and 1 CMA due to low clinical volume.

**Fig. 1:**
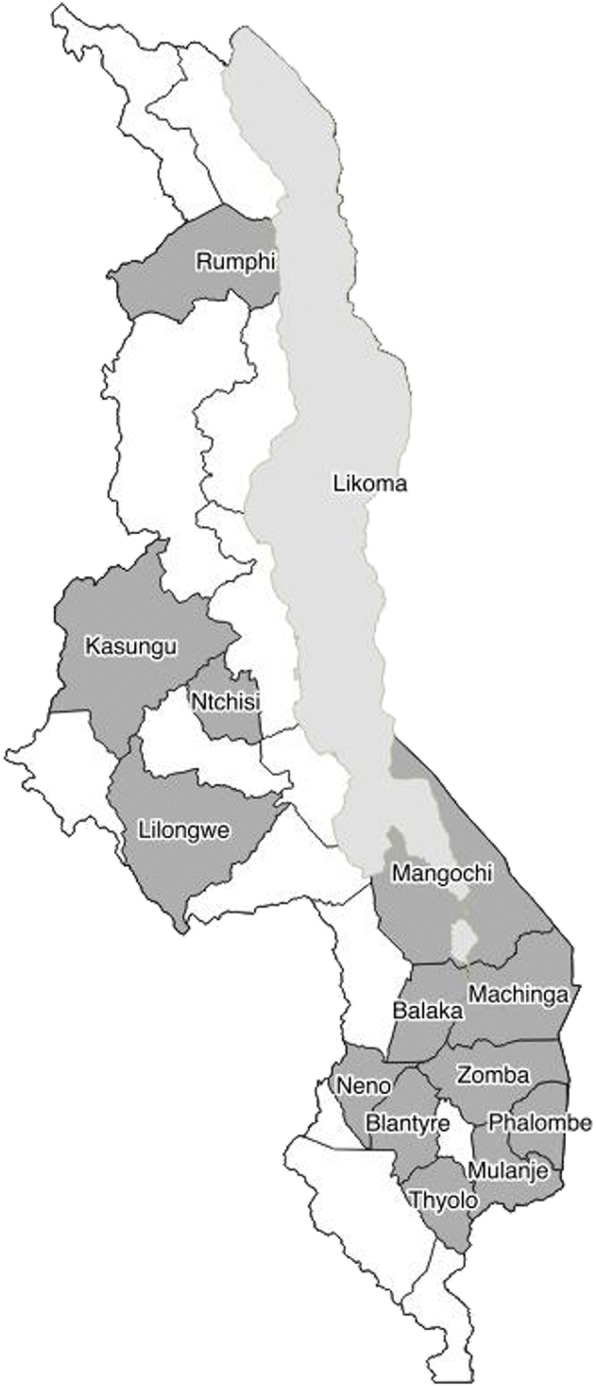
Study district distribution of Malawi. One CMA and one matched NMT from 31 health centers across Malawi were selected for training in Malawi’s 1-week LARC insertion course, of which 29 CMAs and 29 NMTs were used for our study analysis. After the training, the CMAs and NMTs returned to their assigned health center across Malawi. The location of these health centers are highlighted in grey, to illustrate the distribution of our study

The CMAs and other nurse providers came from 14 of the 28 districts and from all 3 regions of Malawi (Table 1, Fig. 1). The CMAs were from a younger age group, with almost half below 24 years of age, whereas the NMTs were older in age, with over half between 24 and 30 years of age (*p* = 0.042). The CMAs were recent graduates, with 96.6% having graduated less than a year ago. In contrast, the NMTs had spent more time in the workforce, with only 24.1% graduating less than 1 year ago (*p* < 0.001).

**Table 1:** Community Midwife Assistant and Nurse Midwife Technician characteristics

Characteristics (*N* = 58)		CMA (*N* = 29)		NMT (*N* = 29)	Fisher’s Exact Test
Age					0.042
< 24	14	(48.3)	5	(17.2)	
25–30	11	(37.9)	16	(55.1)	
> 32	4	(13.8)	8	(27.6)	
District					1.000
North Rumphi, Likoma	5	(17.2)	5	(17.2)	
Central Lilongwe, Kasungu, Ntchisi	10	(34.5)	10	(34.5)	
South Blantyre, Zomba, Mulanje, Machinga, Balaka, Thyolo, Neno, Phalombe, Mangochi	14	(48.3)	14	(48.3)	
Years since graduation from school					< 0.001
< 1 year	28	(96.6)	7	(24.1)	
1–5 years	1	(3.5)	9	(31.0)	
5–10 years	0	(0.0)	9	(31.0)	
11–20 years	0	(0.0)	1	(3.5)	
21+ years	0	(0.0)	3	(10.3)	

*CMA* Community Midwife Assistant, *NMT* Nurse Midwife Technician, *LNG* levonorgestrel

Prior to the LARC training, 55% of the CMAs had never placed a LNG implant, whereas only 28% of NMTs had never placed a LNG implant (*p* = 0.024, Table 2). During the LARC training, 97% of the CMAs at least placed 1 LNG implant, but only 76% of the NMTs placed at least 1 implant (*p* = 0.037).

**Table 2:** Community Midwife Assistant and Nurse Midwife Technician LNG implant insertion statistics

Characteristics (*N* = 58)	CMA (*N* = 29)	NMT (*N* = 29)	Fisher’s Exact Test
#LNG implant placed prior to training			
0	16 (55.2)	8 (27.6)	0.024
1–10	8 (27.6)	8 (27.6)	
11–20	4 (13.8)	3 (10.3)	
21–100	0 (0.0)	6 (20.7)	
> 100	1 (3.5)	4 (13.8)	
#LNG implant placed during training			
0	1 (3.5)	7 (24.1)	0.037
1	13 (44.8)	8 (27.6)	
2	6 (20.7)	8 (27.6)	
3	6 (20.7)	1 (3.5)	
4	3 (10.3)	3 (10.3)	
5	0 (0.0)	2 (6.9)	
#LNG implant placed since training			
0–10	6 (20.7)	7 (24.1)	0.785
11–20	13 (44.8)	15 (51.7)	
> 21	10 (34.5)	7 (24.1)	
Mean LNG implant insertion score			
< 80%	0 (0.0)	2 (6.9)	0.088
81–85%	3 (10.3)	2 (6.9)	
86–90%	10 (34.5)	8 (27.6)	
91–95%	15 (51.7)	10 (34.5)	
96–100%	1 (3.5)	7 (24.1)	

There was no significant difference between CMA and NMT mean LNG implant scores during the monitoring visit (*p* = 0.088). 89.7% of CMAs had an average LNG implant insertion score above the MoH competency score of 85%, while 86.2% of NMTs had an average LNG implant insertion score above the MoH competency score of 85%

### LNG implant insertion scores

After the LARC training, a majority of the CMAs (79.3%) and NMTs (75.8%) had placed at least 11 LNG implants at their health center (*p* = 0.785) by the end of their monitoring visit (Table 2). Overall, CMAs inserted a total of 539 LNG implants, and NMTs inserted a total of 488 LNG implants.

There was no significant difference between CMA (90%) and NMT (86%) mean LNG implant scores during the monitoring visit (*p* = 0.088). Both cadres scored above the MoH competency criterion of 85%. However, 7% of the NMTs scored below 80% (compared to 0% of CMAs) which is well below the competency level. In fact, these 2 NMTs never achieved competency as all 5 of their scores were below 85%. Thirteen (44.8%) of the CMAs and 10 (34.5%) of the NMTs had a score of < 85% on their first implant insertion during the monitoring visit, and all but the 2 NMTs were able to achieve competency within 1–3 implant insertions during the visit.

The most commonly-missed pre-insertion items on the checklist (Appendix A) were: 1) asking about allergies, 2) explaining the benefits and limitations of all contraceptive methods available, 3) asking about reproductive goals, and explanation of possible implant side effects and other health problems. The most commonly-missed items during the implant insertion were: 1) washing and drying hands (mostly due to lack of running water at the health facilities), and 2) not using sterile surgical drapes on the arm (again, due to lack of availability). Finally, the most commonly-missed post-insertion items were: 1) decontaminating the needle and syringe, 2) washing and drying hands, and 3) counselling the client about wound care.

The mean LNG implant score for the CMAs was 90.2%, versus 89.7% for the NMTs, which gave a non-inferior mean percent difference of − 0.5% (95% CI: -3.4%, 2.4%, Table 3). We also found a non-inferior mean percent difference in our adjusted model (1.3%, 95% CI: -2.2%, 4.8%).

**Table 3:** Difference in Mean Percentage of Observation Scores by Nurse Type

Mean % of Observational Scores	NMT	CMA	Difference*	95% CI
	*N* = 29	*N* = 29	β	(Lower CI, Upper CI)
Unadjusted	90.7	89.2	−1.5	−4.4, 1.4
Adjusted**	90.5	89.4	−1.1	−5.0, 2.7

The non-inferiority margin (∆) was set at −10 for this study. While the upper limits of the confidence interval are not interpreted for non-inferiority analyses, the lower confidence interval was interpreted and was found to be within the 10%. The mean difference between the CMAs and NMTs was also found to be within 10%

*Significant at *p* < 0.05

**Scores adjusted or number of insertions pre-training, number of insertions during training, and years since graduation

## Discussion

In our study, CMAs were non-inferior to NMTs with the insertion of LNG implants. We observed that both CMAs and NMTs were generally competent with LNG implant insertion, but it was concerning to find that 10% of CMAs and 14% of NMTs had a mean LNG implant insertion score below competency. With the increase in demand for LARC, particularly implants, in Malawi and other sub-Saharan African countries, [7–10] this study provides evidence that LNG implant insertion may be safely task shifted to Auxiliary Nurse Midwives, such as CMAs, as a way of meeting a country’s FP needs. However, 40% of the nurses had initial LNG implant scores below competency, although most were able to achieve competency within a few monitored insertions. Therefore, we recommend that all nurses undergo continued mentorship and monitoring with LNG implant insertion after the initial training, particularly if they place less than 5 LNG implants during the actual training. Additionally, based on the most commonly missed pre-insertion, during insertion, and post-insertion items we recommend the improvement of reliable running water, and the availability of medical items such as sterile surgical drapes from an infrastructure standpoint.

To our knowledge, this is the first published comparative study that evaluates the competency level of Auxiliary Nurse Midwives with another cadre in LNG implant insertion. A study from Ghana also evaluated the competency level of a similar cadre of providers, called Community Health Nurses (CHNs), to insert and remove LNG implants [11]. CHNs receive 2 years of training and were previously only allowed to counsel and refer for LARC. However, in 2008, a carefully selected group of 33 CHNs across Ghana were trained to insert and remove LNG implants. They were later assessed for their skills. While more than 75% of the CHNs reported no complications with implant insertions, more than 80% of the CHNs and their managers felt that there was a need for further training in infection prevention, counseling, and management of side effects and complications. This study was similar to ours in that it specifically evaluated insertion of LNG implants among an Auxiliary health provider cadre; however, our study compared our cadre’s competency level to a higher cadre to ensure that the two cadres were providing similar levels of care.

Other studies have evaluated the competency levels of Lay Health Worker cadres to insert implants. A study completed in northern Nigeria evaluated the competency of 166 Community Health Extension Workers (CHEWs) to insert both LNG and ENG implants after a 2- or 3-week training [12]. The study required that the CHEWs have at least 15 observed implant insertions before they could proceed to insert implants under supervision on actual clients. Most CHEWs reached competency after insertions on 4–5 actual clients. The authors concluded that task shifting implant insertion is a plausible option to fulfill the family planning needs in Nigeria. However, similar to our study, they also suggested that adequate training and supportive supervision was necessary to ensure high-quality implant insertion services.

### Strengths and limitations

Our study had a few limitations. First, the CMAs and NMTs were evaluated over a 5-month period from November 2016 to March 2017, so the nurses who were evaluated closer towards the end of the period may have had higher scores as they may have had more implant insertions prior to evaluation. However, since the matched CMAs and NMTs for each health center were evaluated at the same visit, and we used mean average scores to compare the two groups, the potentially higher scores at the end should have been balanced out by the potentially lower scores of those CMAs and NMTs who were evaluated towards the beginning of the monitoring period. In addition, in our non-inferiority comparison, we adjusted for the number of LNG implants placed after the training.

Furthermore, our evaluations were completed by FP Master Trainers who were not blinded as to who was a CMA and who was an NMT since CMA and NMTs wear different uniforms at work. Therefore, our Master Trainers (who are both NMTs) may have been subject to bias during evaluation as they may have expected the NMTs to score higher, and the CMAs to score lower. However, we do not believe this occurred as there was a wide range of scores for both the CMAs and NMTs, and there was no difference in their mean average scores.

Despite these limitations, our study has a number of important strengths. As noted earlier, this study is the first to train and evaluate both Auxiliary Nurse Midwives and a higher cadre concurrently to assess and compare their competency levels for LNG implant insertion. In addition, we matched each CMA to another nurse working at their same health center so that we could try to control for the conditions of their health center and the population characteristics of its catchment area. We were adequately powered to analyze our primary outcome and achieved our sample size with minimal loss to follow up. Finally, we were able to evaluate CMAs and NMTs based in all 3 regions of Malawi and 14 of Malawi’s 28 districts.

## Conclusion

Both CMAs and NMTs generally demonstrated competency with the insertion of LNG contraceptive implants after their training, even after adjusting for the years since graduation and the number of implants place. However, almost half of the CMAs and over a third of the NMTs were not competent at the time of their first evaluated LNG implant insertion, and 2 NMTs failed to reach competency within 5 evaluated insertions. Therefore, continued mentorship and supportive supervision after training is critical to ensure that women are receiving high-quality and safe implant insertion services. Future studies could evaluate long-term client satisfaction and continuation with LNG insertion by Auxiliary Nurse Midwives or target other LARC outcomes that we were not powered to analyze. In addition, more comparative studies, such as this one, need to be done so that policymakers have the evidence they need to upgrade the family planning recommendations for various cadres of contraceptive providers.

## Additional file

Additional file 1: Ministry of Health approved Levonorgestrel Implant Insertion Checklist. Two Family Planning Master Trainers visited the nurses’ health centers over a 5-month period and used this Malawi LNG implant insertion checklist to evaluate the first five LNG implant insertions. (DOCX 1561 kb)

## Abbreviations

CHEW: Community Health Extension Worker
CHN: Community health nurse
CMA: Community Midwife Assistant
ENG: Etonogestrel
FP: Family planning
HEW: Health extension workers
HSA: Health Surveillance Assistants
LARC: Long-acting reversible contraception
LNG: Levonorgestrel
NMT: Nurse Midwife Technician
WHO: World Health Organization
