# Supplementary material for: Task shifting levonorgestrel implant insertion to community midwife assistants in Malawi: results from a non-inferiority evaluation
Source: Contracept Reprod Med. 2018 Nov 1;3:24. doi: 10.1186/s40834-018-0077-6 (PMC6211513; doi:10.1186/s40834-018-0077-6)
Supplement: Supplementary file 1 [file 40834_2018_77_MOESM1_ESM.docx]

PRACTICE CHECKLISTS FOR IMPLANTS COUNSELING AND CLINICAL SKILLS

USING THE PRACTICE CHECKLISTS

The **Practice Checklists for Implants Counseling and Clinical Skills** are derived from the information provided in the learning guides. As the participant progresses through the course and gains experience, dependence on the detailed learning guides decreases and the Practice Checklists may be used in their place. The **Practice Checklists for Implants Counseling and Clinical Skills** focus only on the key steps in the entire procedure, and can be used by participants, when providing services in a clinical situation, to rate one another's performance. The practice checklists are the same as the Checklists for Implants Counseling and Clinical Skills which the clinical trainer will use to evaluate each participant's performance at the end of the course. The rating scale used is described below:

CHECKLIST FOR IMPLANTS COUNSELLING AND CLINICAL INSERTION SKILLS
(To be used by Instructor)

Student's Name: ____________________ Dates: ____________

Year of Study: ____________ Facility Name:________

Rate the performance of each step or task observed using the following rating scale:

1. **Needs Improvement:** Step or task not performed correctly or in sequence (if necessary) or is omitted

4. **Competently Performed:** Step or task performed correctly in proper sequence (if necessary) but participant does not progress from step to step efficiently

3 **Proficiently Performed:** Step or task efficiently and precisely performed in the proper sequence (if necessary)

Not Observed: Step or task or skill not performed by participant during evaluation by clinical trainer

CHECKLIST FOR IMPLANTS COUNSELING AND INSERTION SKILLS					
TASK/ACTIVITY	CASES				
1. Greets client and establishes rapport					
2. Reviews client record					
3. Explains the procedure to the client					
4. Asks about allergies					
**PRE INSERTION COUNSELLING**					
Provides general information about the contraceptive methods available:<br>· Explains what that method is<br>· Explains how the method works and its effectiveness<br>· benefits and limitations of each:<br>· Explains possible side effects and other health problems<br>· Explain the most common side effects					
**Method-Specific Counseling**					
1. Obtains biographic information (name, address, etc.)					
2. Asks the client about her reproductive goals (Does she want to space or limit births?) and need for protection against STIs.					
3. Explores any attitudes or religious beliefs that either favor or rule out one or more methods.					
4. Discusses client's needs, concerns and fears in a thorough and sympathetic manner.					
4. Helps client begin to choose an appropriate method.					
**If Client Chooses Implants:**					
5. Screens client carefully to make sure there is no medical condition that would be a problem **(complete Client Screening Checklist).**					
6. Gives the woman specific information about the chosen method:<br>· Shows where and how the method is used<br>· Explains how the method works and its					

CHECKLIST FOR IMPLANTS COUNSELING AND INSERTION SKILLS					
TASK/ACTIVITY	CASES				
effectiveness<br>• Explains possible side effects and other health problems<br>• Explains the most common side effect					
7. Opens sterile implants package by pulling apart sheets of the pouch and dropping capsules into a sterile bowl or tray.					
8. Washes and dries hands appropriately					
9. Puts on sterile gloves					
10. Arranges instruments and supplies on sterile tray.					
11. Counts the number of capsules					
12. Cleans the incision area					
13. Places sterile surgical drape over arm.					
14. Using a sterile needle and syringe, injects local anesthetic (without epinephrine).					
15. Advances needle about 4 cm and as the needle are withdrawn, slowly injects local anesthetic in each of three sub-dermal tracks.					
16. Withdraws needle and places in a safe area					
17. Gently massages the skin					
18. Tests incision site with tip of forceps for adequate anesthesia					
19. If client feels pain, waits 2 minutes and retests incision site.					
INSERTION					
1. Makes a shallow, 2 mm incision with a scapel just through the skin at insertion site. Alternatively, inserts trocar directly through the skin without making an incision.					
2. Inserts trocar and plunger through the incision at a shallow angle with the bevel facing up.					
3. While tenting the skin, slowly and smoothly advances trocar and plunger towards mark (1) nearest the hub on the trocar. The tip of the trocar should be under the first mark on the skin.					
4. Removes plunger from the trocar.					
5. Loads first capsule into trocar using either the gloved thumb and forefinger of one hand or a forcep,					

CHECKLIST FOR IMPLANTS COUNSELING AND INSERTION SKILLS					
TASK/ACTIVITY	CASES				
keeping the other hand cupped beneath the trocar in order to catch the capsule if it falls.					
6. Reinserts plunger and pushes capsule toward tip of trocar just until resistance is felt.					
7. Holds plunger firmly in place with one hand and slides trocar out of incision until it reaches plunger handle.					
8. Withdraws trocar and plunger together until mark (2) leaving the tip of the trocar just near the incision (does not remove trocar from incision.)					
9. Moves the tip of the trocar laterally away from the end of the capsule to be sure it is completely free of the trocar.					
10. Avoids damaging the first capsule by holding it out of the path of the trocar with the forefinger and middle finger of the free hand.					
11. Redirects the trocar about 15^0 to follow next mark on the skin; then advances trocar and plunger towards mark (1) nearest the hub on the trocar.					
12. Inserts remaining capsules using the same technique (steps 4-10).					
13. Palpates the ends of the capsules nearest the shoulder to be sure the capsules are placed correctly.					
14. Palpates incision to be sure that all capsules are about 5mm clear of incision.					
15. Removes trocar from incision only after all capsules have been inserted.					
POST-INSERTION TASKS					
1. Presses down on incision with gauzed finger to stop bleeding.					
2. Removes the drape.					
3. Wipes client's arm with alcohol					
4. Brings edges of incision together and closes it with band aid or surgical tape with sterile gauze.					
5. Applies pressure dressing to insertion area and wraps gauze bandage firmly around the arm					

CHECKLIST FOR IMPLANTS COUNSELING AND INSERTION SKILLS					
TASK/ACTIVITY	*CASES*				
6. Decontaminates needle and syringe and disposes them off					
7. Decontaminates instruments					
8. Disposes of waste materials					
9. Decontaminates and disposes of gloves					
10. Washes and dries hands appropriately					
11. Documents procedure and draws position of capsules in the client's record.					
POST-INSERTION COUNSELLING					
1. Instructs client regarding wound care					
2. Discusses what to do if client experiences any problems.					
3. Asks client to repeat instructions.					
4. Answers client's questions.					
5. Observes client for at least 15 to 20 minutes and asks how she feels before sending her home.					
6. Advises client to come after 3-5 days and yearly thereafter.					
7. Gives client next appointment date.					

Student is ☐ is not ☐ competent to provide counselling to family planning clients.

TEACHER'S COMMENTS

____

____

____

____

STUDENT'S COMMENTS

____

____

____

____

TEACHER'S NAME ______________________ SIGNATURE ______________________
